# Adipocytes contribute to tumor progression and invasion of peritoneal metastasis by interacting with gastric cancer cells as cancer associated fibroblasts

**DOI:** 10.1002/cnr2.1647

**Published:** 2022-06-12

**Authors:** Toshihide Hamabe‐Horiike, Shin‐ichi Harada, Kyoko Yoshida, Jun Kinoshita, Takahisa Yamaguchi, Sachio Fushida

**Affiliations:** ^1^ Center for Biomedical Research and Education, School of Medicine Kanazawa University Kanazawa Japan; ^2^ Department of Gastroenterological Surgery, Division of Cancer Medicine, Graduate School of Medical Science Kanazawa University Kanazawa Japan

**Keywords:** adipocytes, cancer‐associated fibroblasts, gastric cancer, peritoneal metastasis

## Abstract

**Background:**

Peritoneal metastasis (PM) is one of the most common causes of noncurative surgery and the most frequent recurrence pattern in gastric cancer (GC). During the process of PM, GC cells detached from primary tumor interact with human peritoneal mesothelial cells (HPMC) overlapped with adipose tissues such as the omentum or mesentery. Although the interaction with HPMC promotes the malignancy of GC, the role of adipose tissues remains unclear.

**Aims:**

We aimed to clarify how adipose tissue are affected by adjacent primary tumors during the expression of adipokines and to elucidate whether GC cells transform adipocytes into CAFs in vitro. In addition, we investigated whether GC cells are affected by adipocytes in their ability to infiltrate.

**Methods:**

We investigated the phenotypic conversion of adipocytes during the malignant process of GC cells in vivo and in vitro. We evaluated the expression levels of adiponectin in the omental adipose tissue of gastric cancer patients by western blotting. Following adipocytes/gastric cancer cells coculture, adipocyte markers, adiponectin receptors, and inflammatory cytokine markers were detected by real‐time PCR and/or western blotting in the single‐cultured and co‐cultured adipocytes; cancer‐associated fibroblast (CAF) markers were detected by immunofluorescence and western blotting in the single‐cultured and co‐cultured adipocytes; invasion assays were performed in single cultured and co‐cultured MKN45 and OCUM.

**Results:**

In omental adipose tissues that are situated close to the primary tumors, the expression of adiponectin tended to decrease in patients with subserosal or serosal invasion. By co‐culturing with GC cells, adipocytes were dedifferentiated and the expression levels of CAF marker FSP1 and inflammatory cytokines, *PAI‐1* and *IL‐6*, significantly increased (*p* < 0.05). Furthermore, GC cells co‐cultured with adipocytes showed enhanced invasion ability.

**Conclusion:**

Our findings suggest that the phenotypic conversion of adipocytes may promote the malignancy of GC in the construction of the cancer microenvironment of PM.

## 1 INTRODUCTION

Gastric cancer (GC) is the fifth most common type of cancer and the third leading cause of cancer‐related deaths worldwide. The recent development of systemic chemotherapy including molecular targeted therapy, has improved the survival rate of patients with GC, with a 5‐years relative survival rate of 65% in Japan.^1^ However, the prognosis of patients with peritoneal metastasis (PM) from GC is extremely poor, even after receiving multidisciplinary therapy, such as preoperative intraperitoneal/systemic chemotherapy,^2^, ^3^ cytoreductive surgery,^4^, ^5^ and hyperthermic intraperitoneal chemoperfusion.^6^, ^7^

We previously reported that human peritoneal mesothelial cells (HPMCs) and bone marrow‐derived fibrocytes contribute to the cancer microenvironment of PM in GC as cancer‐associated fibroblasts (CAFs) which are transformed by interacting with GC cells.^8^, ^9^, ^10^, ^11^ Using a mouse xenograft model, we demonstrated that GC cells have the ability to proliferate and invade by interacting with CAFs, resulting in the development of a fibrous tumor beneath the peritoneal space.^8^, ^9^, ^12^, ^13^ It should be difficult and insufficient to establish fibrous tumors in contribution with just mono layered HPMCs and attached GC cells.^8^, ^12^, ^14^, ^15^ In addition, bone marrow‐derived fibrocytes are not likely to participate in the promotion of cancer microenvironment because chemokines that recruit them are hardly secreted in the early phase.^16^, ^17^, ^18^ Most of the detached GC cells contact with adipose tissues covered with HPMCs, such as omentum or mesentery, and induce morphological change in HPMCs from cobblestone‐like to spindle shape by TGF‐β derived from GC cells.^8^, ^19^, ^20^, ^21^ Thus, GC cells might migrate into adipose tissues through the gap between HPMCs, resulting to interact with not only HPMCs but also adipocytes to establish the cancer microenvironment for PM.

In this study, we aimed to clarify how adipose tissues are affected by adjacent primary tumors during the expression of adipokines and to elucidate whether GC cells transform adipocytes into CAFs in vitro. In addition, we investigated whether GC cells are affected by adipocytes in their ability to infiltrate.

## 2 METHODS

### 2.1 Adipose tissues

The adipose tissues that are situated close to the primary tumor in the depth of invasion with T1 or T3/T4 were obtained from the omentum during surgery with curative intent. The location of T1 tumor was recognized as marked clip nearby tumor using intraoperative X‐ray. Prior to this study, written informed consent was obtained from each patient.

### 2.2 Cell culture

The human GC cell line MKN45, which was derived from a hepatic metastatic tumor showing a moderately differentiated tubular adenocarcinoma (tub2),^22^, ^23^ was obtained from the American Type Culture Collection (Rockville). OCUM, a cell line derived from a human scirrhous GC with high peritoneal‐seeding activity, was provided by the Department of Surgical Oncology of Osaka City University of Medicine.^24^, ^25^ The pre‐adipocyte 3T3‐L1 cell line was obtained from the JCRB Cell Bank. The MKN45 cells were cultured using RPMI1640 (Gibco), while the OCUM and 3T3‐L1 cells were cultured using Dulbecco's Modified Eagle's Medium with high glucose (Life Technologies), supplemented with 10% fetal bovine serum (Nichirei Bioscience Inc.), 100 IU/ml penicillin, and streptomycin (Gibco). Cell lines were seeded in 10‐cm dishes and cultured in 10 ml of medium at 37°C under a humidified atmosphere with 5% CO_2_. The cells were grown until they reached confluence, harvested using 0.25% trypsin/EDTA (Gibco), and suspended in culture medium before use.

### 2.3 Preparation and differentiation of adipocytes

The adipogenesis assay kit (Abcam) was used for 3T3‐L1 preadipocyte differentiation. 3T3‐L1 preadipocytes were cultured in a 6‐well plate (Falcon) using a differentiation induction medium for 3 days and an insulin medium for 7–10 days.

### 2.4 Co‐culture of adipocytes and GC cells

Tumor cells and adipocytes were co‐cultured using a Transwell culture system (0.4‐μm pore size; Corning). A total of 3.0 × 10^5^ GC cells (MKN45 or OCUM) were seeded in the top chamber of the Transwell system in insulin medium and co‐cultured with or without mature adipocytes in a 6‐well plate for 24, 48, and 96 h.

### 2.5 RNA extraction and real‐time polymerase chain reaction

Total RNA was extracted from single‐cultured adipocytes and co‐cultured adipocytes using an RNeasy mini kit (Qiagen) and was treated with an RNase‐free DNase set (Qiagen), according to the manufacturer's instructions. All extracted total RNAs were assessed for degradation using an Agilent 2100 Bioanalyzer (Agilent). RNAs with an RNA integrity number (RIN) of eight or more were used for quantitative polymerase chain reaction (qPCR).^26^ Complementary DNA (cDNA) was generated from RNA using a SuperScript™ IV First‐Strand cDNA Synthesis Reaction (Invitrogen), following the manufacturer's instructions, and the cDNA samples were stored at −20°C before use.

Each mRNA expression level was estimated using PowerUp SYBR Green Master Mix (Applied Biosystems) on an Mx3000P system (Agilent Technologies) and analyzed using the MxPro qPCR software (Agilent Technologies). The primers used are listed in Table 1. The PCR mixtures for each gene contained PowerUp SYBR Green Master Mix, cDNA template, and optimized primer concentrations, diluted to a final volume of 20 μl with nuclease‐free water. Gene expression was normalized to β‐actin expression using the 2^−ΔΔCt^ method.^27^ Each experiment was performed in triplicate and repeated three times.

**TABLE 1:** Real‐time quantitative RT‐PCR primers

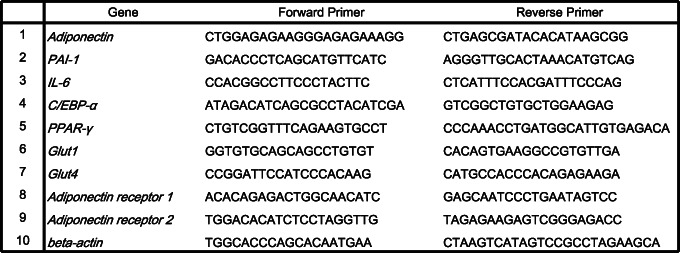

### 2.6 Western blotting

A PRO‐PREP™ kit (iNtRON Biotechnology) was used to extract total proteins from the neighboring omentum of GC patients and from both single‐cultured adipocytes and co‐cultured adipocytes with GC cells. The neighboring omentum of GC patients (100 mg) or the adipocytes were homogenized in 300 μl or 200 μl of PRO‐PREP™ solution by sonication (TOMY). Whole tissues or cell lysates were prepared in denaturing sodium dodecyl sulfate sample buffer, loaded with 5 μg of protein per well in 10% or 4%–20% Mini‐PROTEAN TGX™ Precast Gel (Bio‐Rad), and separated by sodium dodecyl sulfate‐polyacrylamide gel electrophoresis (SDS‐PAGE). Proteins were transferred to polyvinylidene fluoride (PVDF) membranes (Bio‐Rad) using the Trans‐Blot Turbo system (Bio‐Rad). For the assessment of S100A4/FSP‐1, αSMA and β‐actin levels, after blocking with the Can Get Signal® blocking reagent (Toyobo) for 60 min at room temperature, incubated with the primary antibodies in Can Get Signal® solution 1 (Toyobo), and washed with Tris‐buffered saline containing 0.05% Tween‐20 (TBS‐T). Then, the membranes were incubated with secondary antibodies in Can Get Signal® solution 2 (Toyobo). After incubation with secondary antibodies, the antibody–antigen complexes were detected with a LightCapture system (Atto) using an ECL western blotting detection kit (GE Healthcare). To determine the adiponectin and β‐actin levels of the omentum obtained by GC patients and cell lysates, we used two‐color western blotting detection with infrared (IR) fluorescence. After SDS‐PAGE, the proteins were transferred to low‐fluorescence PVDF membranes (Bio‐Rad). After blocking with Odyssey Blocking Buffer (LI‐COR Biosciences) for 60 min at room temperature, incubated with primary and secondary antibodies according to the manufacture's protocol (LI‐COR Biosciences). After incubation with secondary antibodies, IR signals were detected using the Odyssey Infrared Imaging System (LI‐COR Biosciences). The antibodies used here were as follows: anti‐S100A4/FSP‐1 (rabbit polyclonal IgG, diluted 1:500, Abcam), anti‐alpha‐smooth muscle actin (αSMA; mouse monoclonal IgG2a, diluted 1:1000, eBioscience), anti‐adiponectin (rabbit polyclonal IgG, diluted 1:500 for human specimen and 1:2000 for mouse sample, GeneTex), anti‐β‐actin (mouse monoclonal IgG, diluted 1:10000, Sigma‐Aldrich), IRDye® 680LT Goat anti‐Rabbit (diluted 1:20000, LI‐COR Biosciences), and IRDye® 800CW Goat anti‐Mouse IgG (diluted 1:5000, LI‐COR Biosciences).

### 2.7 Immunocytochemistry and fluorescent probe

All cells were fixed in 4% paraformaldehyde solution for 10 min at room temperature. After blocking with PBS containing 0.3% Triton™ X‐100 (PBS‐T) containing 2% skim milk for 90 min, the cells were incubated overnight with primary antibodies in PBS‐T containing 2% skim milk at 4°C. The cells were washed three times with PBS‐T and incubated with secondary antibodies and Hoechst 33342 in PBS‐T containing 2% skim milk at room temperature for 2 h. After washing three times with PBS‐T, the cells were mounted in Mowiol mounting medium (Calbiochem). The antibodies used were as follows: anti‐S100A4/FSP‐1 (rabbit polyclonal IgG, diluted 1:500, Abcam) and anti‐αSMA (mouse monoclonal IgG2a, diluted 1:1000, eBioscience). BODIPY® lipid probe (molecular probes) was used at a concentration of 0.35 mg/ml.

### 2.8 Microscopy

Epifluorescence microscopy was performed using a BZ‐X710 microscope (Keyence).

### 2.9 Invasion assay

After a single culture or co‐culture with adipocytes, GC cells were trypsinized, and 1.0 × 10^4^ cells were added to the inner invasion chamber (BD Bioscience) in a volume of 500 μl with serum‐free medium. The outer wells contained 500 μl of culture medium containing 10% FBS. After 48 h of incubation, non‐invading cells were removed from the inner wells using a cotton swab, while invading cells were fixed with methanol/acetone (1:1) for 10 min and stained with hematoxylin for 10 min. The number of invading cells was counted in 10 different microscopic fields under a 40× objective. This invasion assay was repeated three times for single culture and 96 h co‐culture.

### 2.10 Statistical analysis

The values in graphs represent the mean ± SEM. An unpaired Student's *t*‐test was used to determine the statistical significance. A *p*‐value of <0.05 was considered significant. “*n*” indicates the number of specimens or experiments. All statistical analyses were performed using Microsoft Excel.

## 3 RESULTS

### 3.1 Phenotypic change of adipocytes presents in GC patients

**FIGURE 1:**
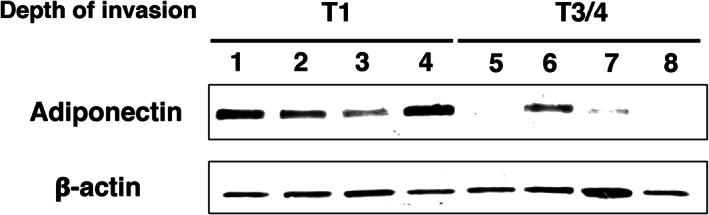
Phenotypic change of adipocyte is present in GC patients. Western blot analysis of the protein expression level of adiponectin in the omentum of GC patients. The protein expression level of adiponectin was decreased in T3/4 patients compared with T1 patients (*n* = 10, both T1 and T3/4, respectively)

We initially examined whether the peritoneal adipose tissue of GC patients has a phenotypic change dependent on the depth of primary tumor invasion in the stomach. To address this issue, a western blotting analysis was performed in the neighboring omentum of patients with GC. The protein expression level of adiponectin was lower in patients with T3/4 disease than in those with T1 disease (Figure 1), although macroscopic PM was not found in these patients. These data suggest that the omental adipose tissue of GC patients exhibited a phenotypic change depending on the depth of primary tumor invasion in the stomach.

### 3.2 Adipocytes transforming into CAFs after co‐culturing with GC cells

**FIGURE 2:**
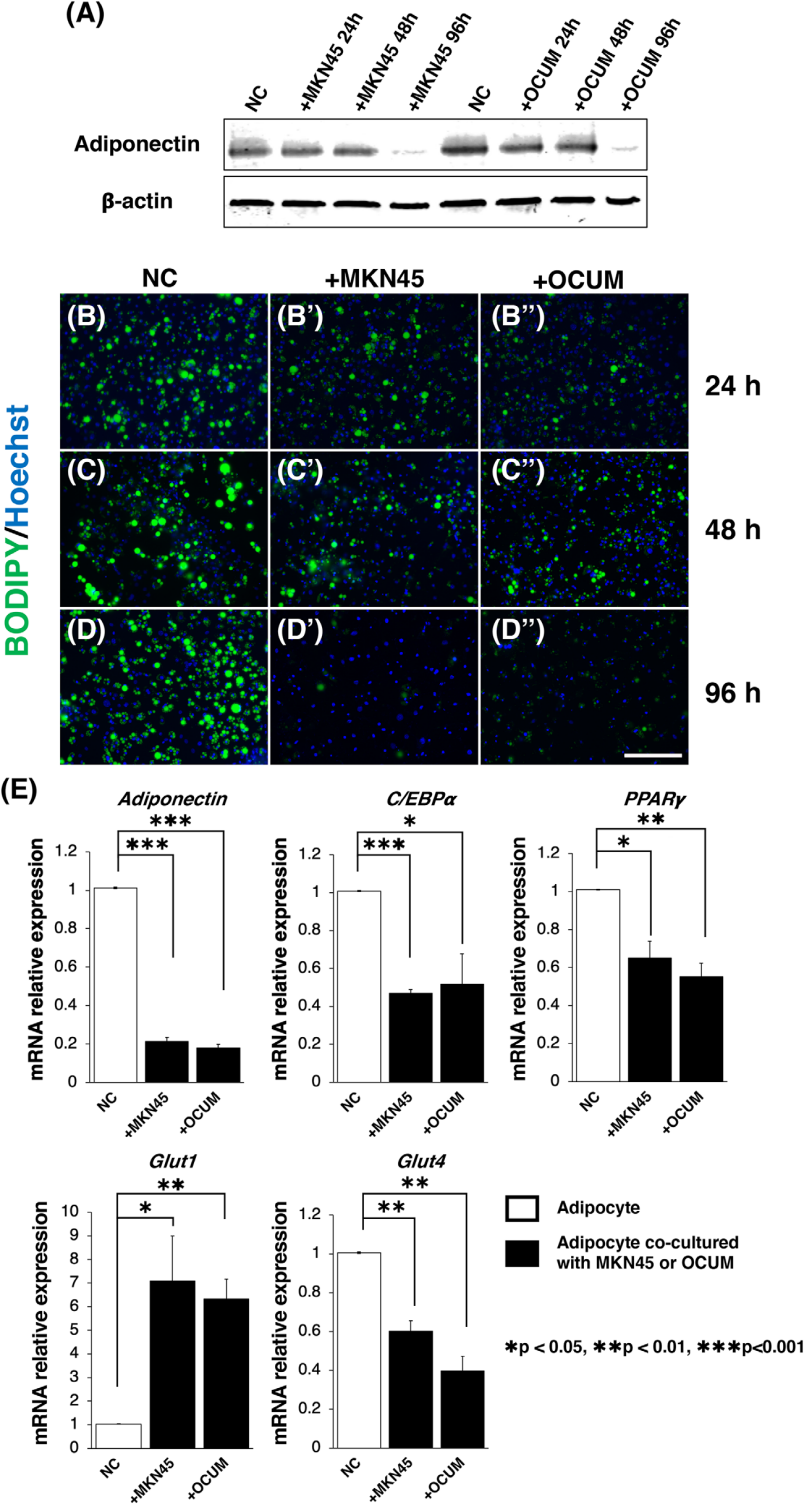
Adipocytes were dedifferentiated by the interaction with GC cells. (A) Western blot analysis of the protein expression level of adiponectin in single‐cultured adipocytes (NC) and co‐cultured adipocytes with GC cells. The protein expression level of adiponectin was decreased in 96 h co‐cultured adipocytes. (B–D) Single‐cultured adipocyte stained with BODIPY and Hoechst 33342. (B′–D′) Adipocytes co‐cultured with MKN45 stained with BODIPY and Hoechst 33342. (B″‐D″) Adipocytes co‐cultured with OCUM stained with BODIPY and Hoechst 33342. The number and size of BODIPY‐positive lipid droplets were decreased in the adipocytes co‐cultured with GC cells for 96 h. (E) Real‐time PCR of adipocyte markers (*adiponectin*, *C/EBPα*, *PPARγ*, and *Glut4*) and pre‐adipocyte marker (*Glut1*) in single‐cultured adipocytes (NC) and co‐cultured adipocytes with GC cells for 96 h. The expression levels of adipocyte markers were decreased, and the expression level of pre‐adipocyte marker *Glut1* was increased in the adipocytes co‐cultured with GC cells for 96 h (*n* = 3, mean ± SEM; two‐tailed Student's *t*‐test, *p < 0.05, **p < 0.01 and ***p < 0.001). Scale bar: 200 μm (B–D″)

A recent study reported that GC cells interact with adipocytes, and this interaction resulted in the malignancy of GC cells.^20^ However, the factors that induced the malignancy of GC cells derived from adipocytes have not yet been sufficiently investigated. Thus, we examined whether we could replicate the microenvironment that can reduce the expression of adiponectin in omental adipocytes of T3/4 GC patients and induce the malignancy of GC cells using an in vitro co‐culture method. Human GC cells, MKN45 and OCUM, were cultured on Transwell plates with or without murine mature 3T3‐L1 adipocytes. To determine whether the phenotypic change of adipocytes was replicated using this co‐culture method, we conducted a western blotting analysis for adiponectin in single‐cultured adipocytes or co‐cultured adipocytes with GC cells. We found that the expression level of adiponectin was reduced after 96 h of co‐culturing with GC cells (Figure 2A).

Subsequently, we hypothesized that GC cells initially induce the malignant progression of adipocytes and then result in the malignancy of GC cells, such as breast cancer cells^28^, ^29^ To clarify this issue, we examined the phenotype of the adipocytes co‐cultured with GC cells. First, to assess the number and size of lipid droplets in adipocytes, we conducted a BODIPY staining of single‐cultured adipocytes or co‐cultured adipocytes with GC cells. We found that adipocytes co‐cultured with GC cells for 96 h exhibited a decrease in the number and size of lipid droplets (Figure 2B–D″). In addition, results of real‐time PCR showed that the expression levels of adipose markers, *adiponectin*, *C/EBPα*, *PPARγ*, and *Glut4* reduced in adipocytes co‐cultured with GC cells for 96 h, respectively (Figure 2E, in both cell lines, *p < 0.05, **p < 0.01, ***p < 0.001). In contrast, the expression level of pre‐adipocyte marker *Glut1* was significantly increased in adipocytes co‐cultured with GC cells for 96 h (Figure 2E, in both cell lines, **p < 0.001). These results strongly suggest that adipocytes co‐cultured with GC cells lose their adipocytic phenotype.

**FIGURE 3:**
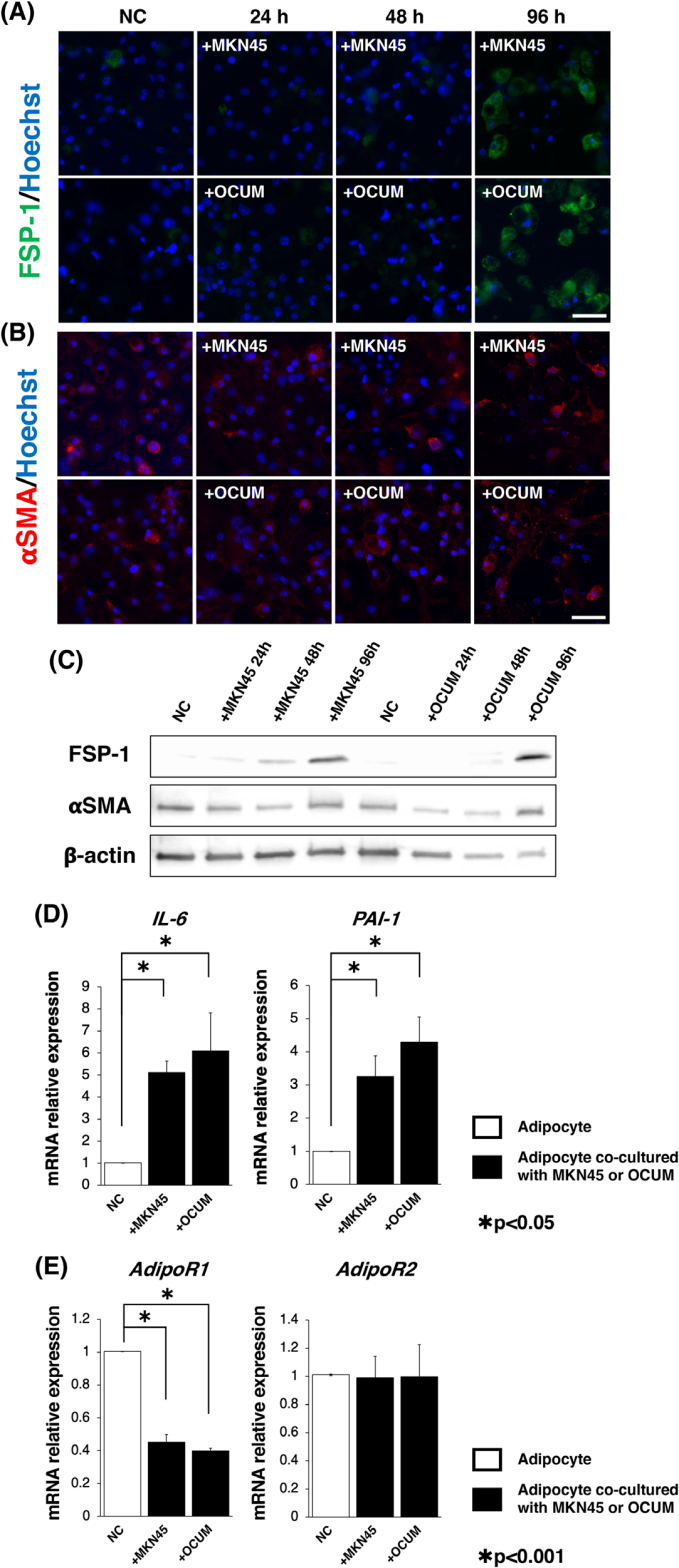
Adipocytic phenotype was converted to that promotes malignancy of GC cells. (A) Representative images of immunocytochemistry for FSP‐1 in single‐cultured adipocytes and co‐cultured adipocytes with GC cells (top; +MKN45, bottom; +OCUM). The expression level of FSP‐1 was increased in the adipocytes co‐cultured with GC cells for 96 h (Green fluorescence). (B) Representative images of immunocytochemistry for αSMA in single‐cultured adipocytes and co‐cultured adipocytes with GC cells (top; +MKN45, bottom; +OCUM). The expression level of αSMA remained low in the adipocytes co‐cultured with GC cells (Red fluorescence). Nuclei (Blue fluorescence) were stained with Hoechst 33342 (A, B). (C) Western blot analysis of protein expression levels of FSP‐1 and αSMA in single‐cultured adipocytes (NC) and co‐cultured adipocytes with GC cells. The result was consistent with each immunocytochemistry in (A) and (B). (D) Real‐time PCR of inflammatory cytokines (*IL‐6* and *PAI‐1*) in single‐cultured adipocytes (NC) and co‐cultured adipocytes with GC cells for 96 h. The expression levels of inflammatory cytokines were increased in the adipocytes co‐cultured with GC cells for 96 h (*n* = 3, mean ± SEM, two‐tailed Student's *t*‐test, *p < 0.05). (E) Comparison of the mRNAs of adiponectin receptors (*AdipoR1* and *AdipoR2*) in single‐cultured adipocytes (NC) and co‐cultured adipocytes with GC cells for 96 h. The expression level of *AdipoR1* was reduced in the adipocytes co‐cultured with GC cells for 96 h (*n* = 3, mean ± SEM, two‐tailed Student's *t*‐test, *p < 0.001). Scale bars: 50 μm (A and B)

Next, to examine the cell type of adipocytes co‐cultured with GC cells after losing the adipocytic phenotype, we examined the expression of major CAF markers, FSP‐1 and αSMA, by immunocytochemistry and western blotting. We found that the expression of FSP‐1 was significantly higher in adipocytes co‐cultured with GC cells for 96 h, but not alter that of αSMA (Figure 3A–C), suggesting that GC cells transformed adipocytes into a CAF‐like cell type. In addition, to examine whether the adipocytes co‐cultured with GC cells possess some factors that can induce malignancy in GC cells, we conducted a real‐time PCR in single‐cultured adipocytes or co‐cultured adipocytes with GC cells for 96 h. We found that the gene expression levels of inflammatory cytokines, *IL‐6* and *PAI‐1*, were significantly increased in the adipocytes co‐cultured with GC cells (Figure 3D, in both cell lines, *p < 0.05).

Furthermore, we explored the mechanisms that increased the gene expression levels of the inflammatory cytokine *IL‐6*. We focused on the fact that the expression level of adiponectin was reduced both in the adipose tissue of T3/4 GC patients and the adipocytes co‐cultured with GC cells (Figures 1 and 2A). A previous study reported that adiponectin signaling suppressed the expression of *IL‐6*.^30^ Thus, we investigated the mechanism that further suppresses the adiponectin signal to increase the expression of inflammatory cytokines in adipocytes co‐cultured with GC cells. qPCR was performed to determine the expression levels of adiponectin receptors; results showed that *adiponectin receptor 1 (AdipoR1)* was reduced in adipocytes co‐cultured with GC cells, but not in *AdipoR2* (Figure 3E, *p < 0.001). This finding suggested that the adiponectin signal might be strongly suppressed by reducing the expression of adiponectin ligand and *AdipoR1* in adipocytes co‐cultured with GC cells. The suppression of adiponectin signaling in adipocytes by interaction with GC cells might promote the expression of *IL‐6*.

### 3.3 Malignancy of GC cells induced by conversion of adipocytic phenotype

**FIGURE 4:**
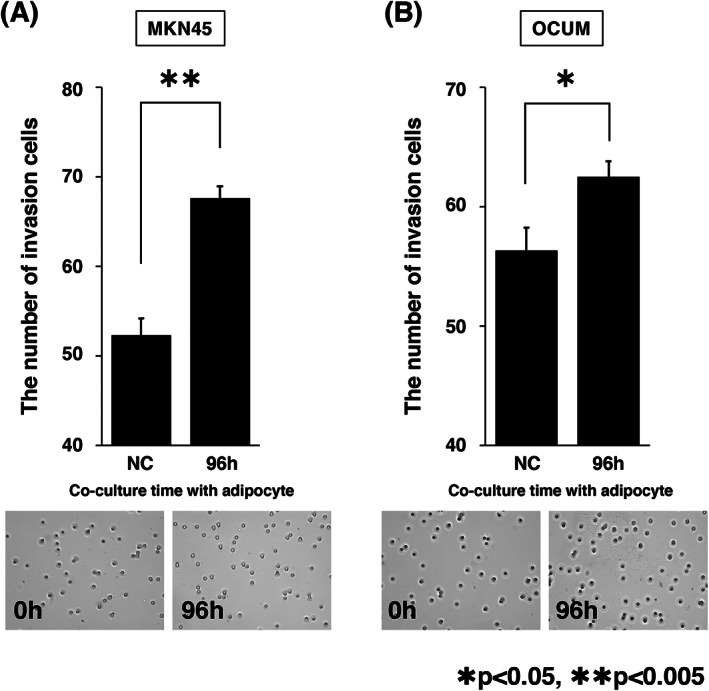
The invasive capacity of GC was increased by co‐culture with adipocyte. (A, B) Matrigel invasion assay of single‐cultured GC cells, co‐cultured MKN45 (A) or OCUM (B) with adipocytes for 96 h. The invasiveness of MKN45 and OCUM were increased by co‐culture with adipocytes compared with single‐cultured GC cells (*n* = 3, mean ± SEM; one‐tailed Student's *t*‐test, *p < 0.05, **p < 0.005)

In addition, to determine whether the malignancy of GC cells is induced by the conversion of the adipocytic phenotype, we conducted a series of Matrigel invasion assays for single‐cultured GC cells or co‐cultured GC cells with adipocytes. We found that the invasiveness of MKN45 and OCUM was increased in GC cells co‐cultured with adipocytes than in single‐cultured GC cells (Figure 4A and B, *p < 0.05, **p < 0.005). These results are consistent with those of a previous study, which demonstrated that the invasiveness of human GC cells, MKN45 and AGS, was enhanced after co‐culturing with adipocytes isolated from the great omentum.^20^ Collectively, the adipocytic phenotype converted to CAF by co‐culturing with GC cells and induced the malignancy of GC cells.

## 4 DISCUSSION

Here we have shown that a phenotypic change of omental adipose tissue is implicated in the malignancy of T3/4 patients. Furthermore, in vitro study, this phenotypic change could be induced by the interaction with GC and the transformation of adipocytes co‐cultured with GC cells into CAFs might promote malignancy of GC cells. This study provides new insight on the interaction between GC cells and adipocytes in the construction of the cancer microenvironment.

The expression level of adiponectin was reduced in omental adipose tissues situated close to the primary tumor of GC patients with subserosal or serosal invasion in the stomach (Figure 1). Although macroscopic PM was not found in these patients, free cancer cells detached from the primary tumor could interact with adipose tissues in the neighboring omentum. In vitro experiments also demonstrated that the decreased expression of adiponectin derived from adipocytes was observed in co‐cultured with GC cells (Figure 2A). We previously reported that adiponectin suppresses the proliferation of GC cells via the AdipoR1 and patients with AdipoR1 showed longer survival than those without AdipoR1.^31^ In addition, continuous infusion of adiponectin into the peritoneal cavity reduced the number of peritoneal metastatic nodules in a mouse model.^32^ Meanwhile, the peritoneal cavity has abundant visceral adipose tissues that produce adiponectin; however, PM is the most common inoperable factor in GC. Our findings that GC cells act on adipocytes to suppress the production of adiponectin resolve this contradiction.

GC cells co‐cultured with adipocytes exhibited increased invasion ability (Figure 4). Previous studies have shown that IL‐6 and PAI‐1 are key factors that enhance the invasiveness of GC cells.^33^, ^34^ Our results were consistent with these reports; the expression levels of *IL‐6* and *PAI‐1* were significantly increased in the adipocytes co‐cultured with GC cells (Figure 3D). There is a possible underlying mechanism that increases the expression levels of *IL‐6* and *PAI‐1*. First, the reduction of adiponectin signals may enhance the expression of *IL‐6*; a previous study reported that the adiponectin signal suppresses the expression of *IL‐6*.^30^ Consistent with this possibility, our results showed that the expression levels of both the adiponectin ligand and the receptor were significantly reduced in adipocytes co‐cultured with GC cells (Figures 2A, E and 3E). Second, TGF‐β derived from GC cells may induce the expression of *PAI‐1*. Previous studies have shown that TGF‐β derived from GC cells induces *PAI‐1* expression in several cells.^35^, ^36^ Importantly, TGF‐β has a potential role in increasing the expression of *PAI‐1* in adipose tissues.^37^ Interestingly, a previous study showed that PPARγ inhibits TGF‐β/ALK5/Smad3‐mediated induction of PAI‐1 in renal mesangial cells.^38^ Our results showed that the expression level of *PPARγ* was reduced in adipocytes co‐cultured with GC cells (Figure 2D) and thus, it suggests that the same signal pathway is involved. Collectively, it is possible that the reduction of PPARγ expression promotes the effect of TGF‐β derived from GC cells to induce *PAI‐1* expression in dedifferentiated adipocytes.

As shown in Figure 3, the adipocytic phenotype was converted to CAF by co‐culture with GC cells. The crosstalk between adipocytes and cancer cells in the microenvironment is well known. Previous studies suggest that in the presence of cancer cells, adipocytes revert from mature, differentiated adipocytes to fibroblast‐like preadipocytes.^28^, ^29^ In addition, we previously reported that HPMCs activated by TGF‐β1 signaling are one of the origins of CAFs.^8^ Therefore, it seems possible that the expression of FSP1/S100A4 may lead to the transformation of adipocytes co‐cultured with GC cells into CAFs through the TGF‐b/Smad pathway.

This study has some limitations. First, the number of clinical samples was relatively small. However, the expression of adiponectin in adipose tissues adjacent to the primary tumors decreased according to the depth of tumor invasion, and crosstalk between adipocytes and cancer cells contributes to tumor progression and invasion. Second, in the absence of vasculature and nutrient supply, metabolic plasticity from glycolysis to lipolysis in cancer cells has not been analyzed. By co‐culturing with GC cells, adipocytes showed dedifferentiation to immature adipocytes, resulting in the reduced expression of *adiponectin*, *PPARγ*, and *C/EBPα* (Figure 2E), which are transcriptional factors involved in adipogenesis and insulin sensitivity.^39^, ^40^ In the dedifferentiated adipocytes, the expression level of *Glut1*, which is a glucose transporter, increased (Figure 2E).^41^ This indicates the conversion of adipocyte energy metabolism from lipolysis to glycolysis in the mitochondria.^40^ In the future, the expression of CD36, which is a mediator of lipid uptake,^42^ should be investigated in various metastatic sites of GC patients, such as lymph nodes, liver, and peritoneal cavity. Third, mouse omentum and mesentery has little adipose tissue, so it is quite difficult to prove interaction between cancer cells and adipocytes in vivo. Moreover, produced peritoneal metastasis does not contain adipose tissue any longer because adipocytes transformed to CAFs. For these reasons, we performed an experiment using clinical tissues to reinforce in vitro data.

**FIGURE 5:**
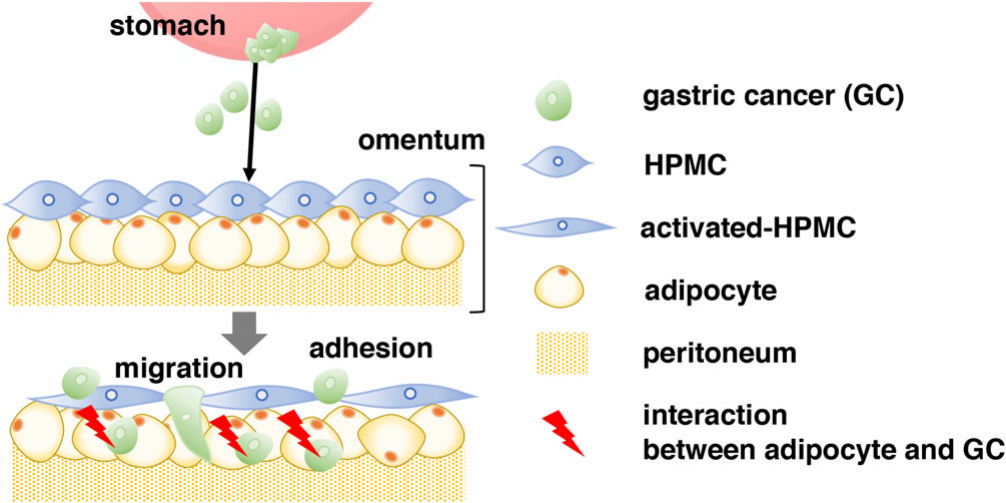
Schematic overview of the interaction between GC cells and adipocytes for PM. The detached GC cells in T3/4 patients might contact neighboring adipose tissue. After GC cells migrate into the gap between HPMCs and subsequently interact with adipocytes to establish a cancer microenvironment for PM

In conclusion, the detached GC cells in T3/4 patients might interact with the neighboring omental adipose tissue, migrate into the gap between HPMCs, and interact with adipocytes to establish a cancer microenvironment for PM, resulting in the induction of a phenotypic change in adipocytes (Figure 5). We demonstrated that dedifferentiated adipocytes not only contribute to tumor progression and invasion but may also be one of the origins of CAFs.

## AUTHOR CONTRIBUTIONS

All authors had full access to the data in the study and take responsibility for integrity of the data and the accuracy of the data analysis. Data Curation, T.H.H, K.Y.; Formal Analysis, T.H.H., K.Y.; Funding Acquisition, J.K., Investigation, T.H.H., S.F.; Methodology, T.H.H., S.H.; Project Administration, S.F., J.K.; Resources, J.K., T.Y.; Software, T.H.H.; Supervision, S.F.; Validation, S.H., T.Y.; Visualization, T.H.H., S.H.; Writing‐Original Draft, T.H.H., S.F.; Writing‐Review & Editing, S.F., S.H.

## CONFLICT OF INTEREST

The authors have stated explicitly that there are no conflicts of interest in connection with this article.

## ETHICS STATEMENT

This study was approved by the Institutional Review Board of the Kanazawa University Graduate School of Medical Science (no. 2278).

## Data Availability

The data that support the findings of this study are available from the corresponding author upon reasonable request.
